# Shared Care for Patients with Diabetes at Risk of Retinopathy: A Feasibility Trial

**DOI:** 10.5334/ijic.4208

**Published:** 2019-09-18

**Authors:** Ranjana Mathur, Dirk F. de Korne, Tien Yin Wong, Donald Tan Tiang Hwee, Peggy P. Chiang, Edmund Wong, Bibhas Chakraborty, Ecosse L. Lamoureux

**Affiliations:** 1 Singapore National Eye Centre, SG; 2 Duke-NUS Medical School, SG; 3 Erasmus School of Health Policy and Management, Erasmus University Rotterdam, Burgemeester Oudlaan, Rotterdam, NL; 4 Ophthalmology and Visual Sciences Academic Clinical Programme, SG; 5 Singapore Eye Research Institute, SG; 6 Camden Medical Centre, SG

**Keywords:** diabetic retinopathy, chronic care management, integrated care, economic benefits

## Abstract

**Purpose:**

While diabetes is a chronic disease, in many health care systems patients with diabetes at risk of diabetic retinopathy (DR) are managed in hospital settings. Aim of this feasibility study is to assess the quality of care and economic benefits of a shared care model managing patients at risk of DR in a primary eye care clinic (PEC) compared with a current tertiary specialist outpatient clinic (SOC).

**Methods:**

A randomized trial was performed, to compare a PEC with a SOC in Singapore. The trial patients included those previously seen at the SOC, and having no DR or stable mild non proliferative (NPDR) with no macular edema, no visual and DR deterioration. Primary outcomes were clinical management. Secondary outcomes were patient satisfaction and cost of consultation. Differences analysis used equivalence testing and generalized odds ratios (GOR).

**Results:**

The trial included 231 patients, 83.1% classified as no DR (PEC: 79.1%; SOC: 87.1%) and 16.9% as stable mild NPDR (PEC: 20.9%; SOC: 12.9%). DR management at PEC was significantly equivalent to that received at the SOC (rate difference 2.56%; CI: (–1.61% to 6.74%)) and 4.29%; CI: (0.14%–8.45%), respectively. Patient satisfaction at the PEC was equally high when compared to SOC (GOR: 1.71; CI: (0.50–2.00)). Direct costs per patient visit was 45% lower at PEC compared to SOC.

**Conclusions:**

Our feasibility trial showed that patients with diabetes with no or stable DR receive similar clinical care and management at a lower-cost PEC setting, are equally satisfied with the service compared to tertiary eye care. A follow-up study is necessary to validate these findings. Managing patients with diabetes at risk of DR at a PEC may be a safe and effective shared care model to improve accessibility for patients while enhancing professional collaboration between hospital and community settings.

## Introduction

Diabetic retinopathy (DR), a condition in people who have diabetes, is the leading cause of vision loss globally, particularly among working age adult population [1,2,3,4,5]. Because it is estimated that more than 200 million people may have DR in the future [3] recent research has been focused on cost effective strategies to care for patients with diabetes at risk of DR while maintaining the quality of care [5]. Most of this research has largely focused on screening for DR, as almost two thirds of diabetic persons have no DR requiring mostly yearly fundus photography or other similar screening modalities [6].

Of patients with DR, only 10 to 15% may have vision-threatening DR (severe non-proliferative DR, proliferative DR, diabetic macular edema (DME)) that requires intensive specialist eye care, including closer follow-up intervals (e.g., 4–6 months) or treatment such as intraocular injection of anti-vascular endothelial growth factor (VEGF), laser therapy or surgery [7,8]. For others with less severe and stable DR, the necessity of being seen in a specialist eye care or tertiary hospital setting is questionable. It is unclear if these patients could have equivalent care by non-specialist at a lower cost in a primary eye care setting. In the Singapore healthcare system, diabetes care is managed at the primary and tertiary levels. Annual fundus photo screening is done at the primary community health service clinics (polyclinics), and patients are referred to tertiary hospital due to poor quality photo, media opacity, small pupil, mild non-proliferative DR (NPDR) and above. The country tops the Organization for Economic Corporation & Development’s (OECD) ranking for diabetes related hospital admissions (430 admissions/100,000 populations; OECD average:160/100,000) [9]. Over the last decades, Singapore’s health care landscape has evolved to favor specialization and tertiary care as funding and health policy have catered to the growth of hospitals and national specialist centers, leaving the primary care sector to free market forces. As public trust in primary care in general is low [10], steps need to be taken carefully and safety nets to be built in any task-shifting activity for low-risk patients. Currently in Singapore, as in many Asian countries, there are legal barriers for optometrists to be involved in follow-up care and decision making. It is usual practice for all eye care (other than dispensing glasses/contact lens) to be delivered by specialists in either hospital-based or private clinics.

While models for primary eye care differ between countries [11,12], treating patients in shared care models between hospital and primary care has in various countries been related to an efficient use of resources, as well as improved health outcomes by promoting positive patient behaviors such as adherence to medication and keeping appointments as patients are more proactively monitored throughout and beyond their cycles of care [13,14,15,16,17]. Studies in eye care have estimated that at least 20% of the tertiary hospital visiting patients only require monitoring [18,19,20]. Findings from other chronic conditions (e.g. diabetes and chronic obstructive pulmonary disease) have shown promising results on substitution of care for stable patients from a hospital to a primary care or home setting [13,14,15].

In this paper, we assessed the feasibility, patient satisfaction, and economic benefits of a new shared care model in Singapore, where patients with diabetes at risk of DR are managed by trained primary care physicians in a primary eye care (PEC) clinic. We hypothesized that the clinical assessment and management of patients at risk of DR and patient satisfaction by primary care physicians trained specifically to manage patients with stable eye conditions at PEC are comparable to the current care at a tertiary specialist outpatient clinic (SOC), with lower costs of consultation at PEC.

## Methods

### Setting

The Singapore National Eye Centre (SNEC) is a public tertiary specialty academic eye hospital. Annually around 300,000 patients visit its SOCs. Around 27,000 (9%) of these outpatient visits are related to DR as the patient was referred to SNEC based on a single retina photograph that was suspected to be abnormal. For this study, SNEC established a primary eye care (PEC) clinic, located 6 km away from the main center. We conducted a randomized equivalence trial comparing the management of 231 patients with diabetes at risk of DR in PEC by trained primary care physicians versus a SOC by resident ophthalmologists.

The study was approved by the institution’s Centralized Institutional Review Board and informed consent was obtained from all patients, in accordance with the Helsinki declaration.

### Clinical Pathway Development and Training Program

Prior to initiation of the study, DR-PEC clinical pathways were drafted by three specialists in the Vitreoretinal department of SNEC (Appendix 2). The pathways were designed based on the principle of referring patients with stable DR to a PEC setting.

*Inclusion criteria:* Patients with **diabetes at risk of DR** were defined as:

– **Under treatment for diabetes (Type I, II) at SOC for at least one visit;**
– No DR but with media opacity < No4, NC4, C5 or P4;
– Stable mild non-proliferative DR with no evidence of diabetic macular edema (DME), vision deterioration, or DR progression for at least two years;
– Stable post-PRP (pan retinal photocoagulation) patients with no evidence of DME, vision deterioration, or DR progression for at least two years.

*Exclusion Criteria:* Patients with:

– Moderate NPDR (non-proliferative DR) or worse;
– Presence of DME;
– Multiple ocular co-morbidities;
– One eyed patient with good eye vision <6/18;
– Small pupil/difficulty in examining the patient.

A training program was developed based on these clinical pathways and protocols. Candidate primary eye care (PEC) physicians were selected from a pool of medical officers who had finished basic medical training and had at least one-year general clinical experience. They received 6 months on the job training by a consultant and were specifically trained in general ophthalmology, which included competency in slit-lamp and indirect ophthalmoscope examination and the interpretation of retinal photos. Although the PEC physician could manage general diabetes, for this particular study his role was to assess the eye management and provide counselling for diabetes control. The costs of the training program consisted mainly of covering the trainee and were around $48,000 per PEC physician.

The medical records of all patients previously on follow-up at the SOC for a duration of more than or equal to 8 months from their last SOC visit (as common practice is to plan a follow-up visit for no DR of mild NPDR after 9 months) were reviewed by medical officers and short-listed according to a list of inclusion criteria as indicated in the clinical pathways protocol before random allocation to either a PEC physician or a resident ophthalmologist for their next visit. Only the study coordinator and one of the co-investigators knew the randomization code. A medical officer who was familiar with the PEC protocols reviewed the notes to shortlist cases (see Appendix 2). All clinicians involved in examining the patients were masked to patient’s previous eye condition and medical records. The coordinator and the co-investigator were present for all patient’s appointments.

On the day of patients’ appointment, the coordinators retrieved the case notes of potential eligible patients, the co-investigator obtained patient’s informed consent, attached a checklist to a new medical record folder. Directly after eye examination by the PEC physician or SOC trainee medical officer, a senior vitreoretinal consultant examined the patient, reviewed the patient’s checklist filled up by the PEC physician or the SOC trainee medical officer, using a dichotomous tick box approach (i.e., correct clinical assessment: yes/no; satisfactory management: yes/no; Appendix 3), which was used as gold standard for the purpose of our research study. Additional explanatory comments or drawings were made when appropriate. The research check list was later merged with the medical records.

At the end of consultation, study coordinators administered questionnaires to the recruited patients. An 11-item questionnaire, being the outpatient segment of an existing widely validated patient satisfaction questionnaire [21], asked patients to rate the following topics: 1) waiting time, 2) interaction with doctor, 3) thoroughness of examination, 4) whether medical care meets expectation, 5) competency of medical staff, 6) whether medical care could be improved, 7) friendly and courteous treatment of doctor, 8) carefulness of checks by staff, 9) all services received, 10) experience at clinic, and 11) overall care received. All answers were rated on a 1–5 Likert scale. 179 questionnaires (response rate of 77%) were completed at the site and collected by the study coordinator. All patients were reimbursed 10 Singapore dollar cash for their travel expenses.

### Consultation costing

Consultation costs of PEC and SOC were determined by the hospitals financial department using the ‘Guidelines for cost-effectiveness research’ [22]. Manpower, direct, and allocated overhead costs were based on the actual workload figures. Consultation manpower costs at SOC were based on the consultant’s sessional rate, while the PEC-consultation was based on the PEC-physician rate. For refraction, SOC used average sessional rate for senior optometrist while PEC used optometrist’s rate. Fundus photography at PEC was performed by optometrists, while senior ophthalmology investigations technologist’s sessional rate was used at SOC, therefore incurring in a higher sessional rate at SOC. Cost of drugs and consumables during consultation were assumed to be similar for PEC and SOC as differences in the actual care delivery were not the focus of this study. Leasing was based on the Singapore Temporary Occupation License (TOL) prizes, which were lower for the PEC premises, with the exception of the lower load of fundus photography.

### Statistical analysis

Equivalence testing was applied to compare the clinical assessment and patient satisfaction rates between the two groups [23,24,25,26]. Assuming an equivalence margin of 10% (PEC rate – SOC rate < 10%), a sample size of 203 was considered adequate. Equivalence testing conclusions, using the upper and lower equivalence margin, were categorized as: superior (better than); non-inferior (at least as good as); equivalent (equivalent to); non-superior (at most as good as) or; and inferior (worse than) [27]. Even though it is less common to report p-values in case of equivalence testing, the conclusions from such testing procedure are based on rigorous statistical significance principles, as opposed to mere empirical observations [25].

Differences in patients’ experiences and satisfaction between PEC and SOC were analyzed using the generalized odds ratio (GOR) [26], an extension of the notion of usual odds ratio for binary data to the case of ordered categorical data (with potential more than two ordered categories), and associated confidence intervals. In the present context, GOR >1 indicates that PEC looks empirically better and GOR <1 indicates that PEC looks empirically worse, but statistical inference can only be drawn in terms of confidence intervals (CI) as follows [27]. If the lower limit of the CI is above the lower limit of the pre-specified equivalence range (0.50), and if the upper limit of the CI is above the upper limit of the pre-specified equivalence range (2.00), then PEC is inferred to be significantly at least as good as (or: non-inferior) the SOC.

Data were analyzed using STATA (Statistics Data Analysis, Stata Corporation, TX, U.S.A.) and R (http://www.r-project.org).

## Results

### Patient characteristics in PEC and SOC

Of the 231 patients, 192 (83.1%) had no DR (PEC: 91 (79.1%); SOC: 101 (87.1%)) and 39 (16.9%) had stable mild non-proliferative DR (PEC: 24 (20.9%); SOC: 15(12.9%)). There were no post-PRP patients with stable DR. All others had no DR. Patients’ characteristics were comparable between the PEC and SOC arms (Appendix 4) with the exception that patients seen at PEC were older (p = 0.013), had a lower rate of Type 2 diabetes mellitus (p = 0.001), a lower monthly income p = 0.002 and used less diabetes related medication (p < 0.001).

### Clinical decision-making at PEC and SOC

At PEC, 97.39% of the patients were provided clinical assessment in concordance with the gold standard (senior vitreoretinal consultant) compared to 94.83% for the SOC (Table 1).

**Table 1:** Comparison of clinical assessment and management of patients with diabetes at risk of DR between PEC and SOC.

	Rate for PEC (%) (n = 115)	Rate for SOC (%) (n = 116)	Diff in Rates (%)	Confidence Interval (%,%)	Equivalence Range (%,%)	Conclusion about PEC*

Correct Clinical Assessment	97.39	94.83	2.56	(–1.61, 6.74)	(–10, 10)	Equivalent
Satisfactory Management	98.26	93.97	4.29	(0.14, 8.45)	(–10, 10)	Equivalent

PEC, Primary Eye Care Clinic; SOC, Specialist Outpatient Clinic. Correct clinical assessment and management is determined through a dichotomous tick box approach (correct clinical assessment: yes/no; satisfactory management: yes/no) according to senior vitreoretinal specialist. * These conclusions are based on rigorous statistical significance principles, and derived from the confidence intervals and the equivalence range for generalized odds ratios.

Patient management was rated as ‘satisfactory’ by the senior vitreoretinal consultant in 98.26% of the patients by PEC physician compared to 93.97% at SOC trainee medical officer. For correct clinical assessment and satisfactory management, the confidence interval for the difference between the two rates (PEC vs. SOC) was entirely contained within the upper and lower limit of the pre-specified 10% equivalence range. This finding suggests that the clinical decision making and clinical management of two services are significantly equivalent.

In 20 cases (8.7%), 7 (6.1%) at PEC and 13 (11.2%) at SOC, there was disconcordance with the gold standard. The potential impact on patients is described in Appendix 5. In 70% of the cases (14 out of 20), the disconcordance had no impact on the patient. In one case at SOC, epiretinal membrane and vitreomacular traction which can potentially have significant impact on the patient was missed.

### Patient satisfaction of PEC and SOC

Differences between PEC and SOC in patients’ experiences and satisfaction are shown in Table 2.

**Table 2:** Comparison of patient satisfaction between PEC and SOC.


Items		Sample Size	Generalized Odds Ratio (GOR)*	Confidence Interval for GOR	Equivalence Range for GOR	Conclusion about PEC**

*1*	*Waiting time to see doctor*	179	1.36	(0.91, 2.02)	(0.50, 2.00)	Non-inferior
*2*	*Interaction with doctor*	179	1.54	(0.94, 2.54)	(0.50, 2.00)	Non-inferior
*3*	*Thorough examination*	124	0.72	(0.33, 1.57)	(0.50, 2.00)	Non-superior
*4*	*Received care met expectations*	179	1.17	(0.66, 2.08)	(0.50, 2.00)	Non-inferior
*5*	*Medical staff appeared competent*	179	1.98	(1.00, 3.93)	(0.50, 2.00)	Non-inferior
*6*	*Medical care can be improved*	179	1.78	(1.21, 2.61)	(0.50, 2.00)	Non-inferior
*7*	*Friendly and courteous doctor*	179	1.60	(0.89, 2.88)	(0.50, 2.00)	Non-inferior
*8*	*Staff carefully checks all*	179	2.27	(1.12, 4.59)	(0.50, 2.00)	Non-inferior
*9*	*Services by all staff*	179	1.89	(1.11, 3.23)	(0.50, 2.00)	Non-inferior
*10*	*Overall care received*	179	0.82	(0.53, 1.27)	(0.50, 2.00)	Equivalent
*11*	*Overall clinic experience*	179	1.71	(1.07, 2.73)	(0.50, 2.00)	Non-inferior


* Generalized Odds Ratio (GOR) > 1 indicates that PEC is empirically better. ** These conclusions are based on rigorous statistical significance principles, and derived from the confidence intervals and the equivalence range for generalized odds ratios.

For 9 of the 11 items, the patients’ experiences at PEC were significantly at least as good as (or: non-inferior) those at SOC. The differences were the largest for the items *‘all staff at this clinic was careful to check everything when treating and examining me’* (GOR: 2.27; CI: (1.12, 4.59)) and *‘the medical staff that treated me appeared competent’* (GOR: 1.98; CI: (1.00, 3.93)). PEC was indicated to be significantly as good (or: non-superior) as SOC on the item *‘the doctor is thorough in examining me’* (GOR: 0.72; CI: (0.33, 1.57)). Finally, when patients were asked the question *‘how would you rate the overall care that you received at this clinic’*, the PEC scores were equivalent to SOC (GOR: 0.82; CI: (0.53, 1.27)). Patients were equally satisfied with the PEC compared to the SOC setting.

### Direct consultation costs of PEC and SOC

The differences in consultation costs between PEC and SOC are reported in Table 3.

**Table 3:** Comparison of the consultation and examinations costs at PEC and SOC.

Cost item	Consultation ($)		Pre-consult Evaluation ($)		Refraction Test ($)		Fundus Photography ($)	

	PEC	SOC	PEC	SOC	PEC	SOC	PEC	SOC

Manpower	19.32	74.08	8.66	8.66	8.63	16.29	5.75	10.63
Drugs & Consumables	0.88	0.88	0.68	0.68	0.11	0.11	1.03	1.03
Leasing	1.37	3.12	0.94	0.00	0.40	1.06	2.28	0.45
Overhead	29.81	99.69	0.00	0.00	17.94	1.37	17.94	2.41
Depreciation	1.13	0.00	1.13	0.19	0.33	1.70	5.54	1.25
**Total costs**	52.51	177.77	11.40	9.53	27.40	20.53	32.55	15.77

Manpower costs at PEC were lower for consultation, refraction test and fundus photography. Overhead costs for consultation were higher for SOC. Depreciation costs were higher for PEC. The total consultation costs at SOC were 3.4 times higher than PEC ($177.77 vs 52.51), while the costs of the pre-consult-evaluation, refraction test and fundus photography were 1.6 times lower at SOC ($71.36 vs. 45.83). Assuming that one patient visit comprises of the pre-consult evaluation, a refraction test, fundus photography and consultation, the total costs of a visit to PEC were estimated to be 44.6% lower than a visit to SOC ($123.87 vs. 223.60).

## Discussion

Our study demonstrates that PEC physicians in a shared care model are able to generate economic benefits by delivering comparable standard clinical DR care to diabetes patients at risk of DR at a primary level compared to current care provided by resident ophthalmologists at a SOC, and that clinical assessment and management are comparable to the gold standard of the senior vitreoretinal consultant for this defined stable DR patient mix. In addition to service delivery at a lower cost at PEC compared to SOC, there was no compromise on patient satisfaction level between centers. These results are promising as currently much of the load of patients with DR in Asia is still considered as sub-acute care, resulting in overcrowded specialty outpatient clinics with substantial waiting times [10]. Our findings have therefore a substantial potential to positively address the economic issues associated with sustainability and overcrowding in the delivery of DR care in tertiary centers in Singapore and other countries with similar healthcare system set-ups.

As the clinical assessment and management are similar, PEC seems to be a promising new model of care for stable DR. In our view, the model is effective due to the close supervision during the training of PEC doctors, a strict assessment on their readiness to start PEC clinics, and an appropriate identification of suitable cases and strict shared care criteria. While the disconcordance rates and potential impact on patients are low, our findings highlight training topics that are important for both medical officers at the SOC as well as primary care physicians. As previous studies have shown disconcordance on clinical assessment, including intra-disconcordance [28], our findings seem to suggest that there is room for further training and standardization in both shared care clinics involved.

The sustainability of PEC is also dependent on the perceived financial and practical benefits for both patients and specialists. The cost differences between PEC and SOC are related to manpower and overhead costs and limited to the local health system. In our study, calculations for manpower costs at SOC were based on the average consultant’s rate. In SOC’s actual practice however, patients are distributed over doctors of different ranks (from medical officers and registrars to senior consultants) using a team-based supervision approach. Overhead costs for consultation at SOC are higher due to the fact that more overhead costs (e.g. human resource, administration and operations). Depreciation costs were higher for PEC due to the lower patient load, with the exception of refraction tests since SOC uses newer automated refraction machines. Since the costs are related to the set-up of the clinic, it is not possible to reduce the overhead costs at SOC substantially without the change to PEC. During our study period, the actual fee that patients paid for consultation was similar at PEC and SOC. It was not feasible to adjust the patient fees during the study due to existing government subsidy schemes and insurance coverage regulations. Taking into account the different ranks of the physician involved, it seems appropriate to consider an adjusted fee for patients at PEC.

An important difference compared to earlier described practices of ophthalmic task substitution is related to the health care system. In contrast to national healthcare systems in Australia, Netherlands and the U.K., Asian health care is more privatized with larger out-of-pocket contributions to be borne by the patients [13,14,15,16,18,19,20]. Patients here (over 50% of the eligible patients refused to enter the study) might have stronger convictions that only the most senior doctor delivers the most appropriate care, and by virtue of them footing out expenses at a SOC which will incur a higher cost compared to primary care, they have a ‘right’ to see a specialist [10]. Related financial and reimbursement schemes will therefore play an important role in the successful implementation of PEC on a larger scale.

Possible incentives for the shared care model include appointment availability for new patients, more time for complex cases and the opportunity for case discussion and education of junior doctors during clinic. At the same time, there is a disparity of generalists delivering chronic care in the Singapore public sector as currently about 86% of general practitioners work in the private sector, but managing only half of the load of chronic conditions [29]. The experiences of Lim et al. with rheumatology shared care in Singapore show that private funding status and partnership with private family physicians is paramount [30]. As long as there is a financial disincentive for both hospitals and primary care, the shared care volumes will be lower than expected on medical criteria only and not move toward even more integrated services with allied health professionals. Currently in Singapore, as in many Asian countries, (legal) barriers for optometrists to be involved in follow-up care and decision making might need to be lowered.

Our pilot study has limitations. Appropriate training of the primary care physicians was a key element in our study setting. As it was the first pilot in Singapore, only one primary care physician contributed to the study, while 28 medical officers at the SOC and 9 senior vitreoretinal consultants were involved. This might limit the generalizability of the study findings and the feasibility of the model. It was possible for the primary care physician to be trained in ophthalmology for six months. While the six months provided a complete and comprehensive curriculum, we do think that this potentially could be compartmentalized and delivered over a different time frame to enable more primary care physicians to join. While the patients in the two arms are comparable, they also differ in a number of aspects. This might have influenced the results; however, as shown in Table 3 there is no direct link to disconcordance found. Also, the patient’s review of the experience might have been slightly artificial as they would usually not get reviewed by the senior vitreoretinal consultant, in addition to their PEC physician.

## Conclusion

In conclusion, PEC is a cost saving, safe, and clinically effective shared care model to improve accessibility for patients while enhancing professional collaboration between hospital and community settings. Patients with diabetes with no or stable DR receive similar clinical treatment at a lower-cost primary eye care setting and are equally satisfied with the service when compared to current tertiary eye care. A longer term follow-up and adherence to the shared care clinical pathway including sensitivity analysis for the cost allocation are currently being evaluated.

## Additional Files

The additional files for this article can be found as follows:

10.5334/ijic.4208.s1 Appendix 1. Recruitment of study patients.

10.5334/ijic.4208.s2 Appendix 2. Clinical Pathways for referral of patients with DR from Specialist Eye Clinic (SOC) to Primary Eye Care Clinic (PEC).

10.5334/ijic.4208.s3 Appendix 3. DR Clinical Diagnosis & Management Checklist.

10.5334/ijic.4208.s4 Appendix 4. Characteristics of patients with diabetes at risk of DR in PEC and SOC.

10.5334/ijic.4208.s5 Appendix 5. Disconcordance in clinical assessment and or management with gold standard: potential impact on patients.

## References

[1] Yau, JWY, Rogers, SL and Kawasaki, R. Global prevalence and major risk factors of diabetic retinopathy. Diab Care, 2012; 35: 556–64. DOI: 10.2337/dc11-1909 PMC3322721 22301125

[2] Zheng, Y, Lavanya, R and Wu, R. Prevalence and causes of visual impairment and blindness in an urban Indian population: the Singapore Indian Eye Study. Ophthalmol, 2011; 118: 1798–804. DOI: 10.1016/j.ophtha.2011.02.014 21621261

[3] Zheng, Y, Lamoureux, EL and Lavanya, R. Prevalence and risk factors of diabetic retinopathy in migrant Indians in an urbanized society in Asia: the Singapore Indian eye study. Ophthalmol, 2012; 119: 2119–24. DOI: 10.1016/j.ophtha.2012.04.027 22709419

[4] Rosman, M, Zheng, Y and Lamoureux, E. Review of key findings from the Singapore Malay Eye Study (SiMES-1). Singapore Med J, 2012; 53: 82–7. 22337179

[5] Cheung, N, Mitchell, P and Wong, TY. Diabetic retinopathy. Lancet, 2010; 376: 124–36. DOI: 10.1016/S0140-6736(09)62124-3 20580421

[6] International Council of Ophthalmology (ICO). Guidelines for Diabetic Eye Care San Francisco, USA: ICO, 2014 Available from http://www.icoph.org/downloads/ICOGuidelinesforDiabeticEyeCare.pdf. Accessed at 13 May 2017.

[7] Ding, J and Wong, TY. Current epidemiology of diabetic retinopathy and diabetic macular edema. Curr Diab Rep, 2012; 12: 346–54. DOI: 10.1007/s11892-012-0283-6 22585044

[8] Shen, ZZ, Huang, YY and Sieh, CJ. Early short-term intensive multidisciplinary diabetes care: a ten-year follow-up of outcomes. Diab Research Clin Pract, 2017; 130: 133–141. DOI: 10.1016/j.diabres.2017.05.022 28618325

[9] Organisation for Economic Collaboration and Development (OECD). Health at a glance. 2015 Available from: http://stats.oecd.org/index.aspx?DataSetCode=HEALTH_STAT. Accessed 22 April 2017.

[10] Khoo, HS, Lim, YW and Vrijhoef, HJ. Primary health care system and practice characteristics in Singapore. Asia Pac Fam Med, 2014; 13: 8 DOI: 10.1186/s12930-014-0008-x 25120380 PMC4129466

[11] Shickle, D, Davey, CJ and Slade, SV. Why is the General Ophthalmic Services (GOS) Contract that underpins primary eye care in the UK contrary to the public health interest? Br J Ophthalmol, 2015; 99: 888–892. DOI: 10.1136/bjophthalmol-2014-305345 25273827

[12] Kovai, V, Rao, GN and Holden, B. An estimate of patient costs and benefits of the new primary eye care model utilization through vision centers in Andhra Pradesh, India. Asia-Pac J Public Health, 2010; 22: 426–435. DOI: 10.1177/1010539510370779 20483829

[13] Coleman, K, Austin, BT, Brach, C and Wagner, EH. Evidence on the chronic care model in the new millennium. Health Aff, 2009; 28: 75–85. DOI: 10.1377/hlthaff.28.1.75 PMC5091929 19124857

[14] Elissen, AM, Steuten, LM, Lemmens, LC, et al. Meta-analysis of the effectiveness of chronic care management for diabetes: investigating heterogeneity in outcomes. J Eval Clin Pract, 2013; 19: 753–62. DOI: 10.1111/j.1365-2753.2012.01817.x 22372830

[15] Rank, MA, Branda, ME and McWilliams, DB. Outcomes of stepping down asthma medications in a guideline-based paediatric asthma management program. Ann Allergy Asthma Immunol, 2013; 110: 354–58. DOI: 10.1016/j.anai.2013.02.012 23622006

[16] Thompson, AC, Thompson, MO and Young, DL. Barriers to follow up and strategies to improve adherence to appointments for care of chronic eye diseases. Invest Ophthalmol Vis Sci, 2015; 56: 4324–31. DOI: 10.1167/iovs.15-16444 26176869

[17] Marahrens, L, Kern, R and Ziemssen, T. Patients’ preferences for involvement in the decision-making process for treating diabetic retinopathy. BMC Ophthalmol, 2017; 17: 139 DOI: 10.1186/s12886-017-0526-z 28793881 PMC5551005

[18] Gray, SF, Spry, PG and Brookes, ST. The Bristol shared care glaucoma study: outcome at follow up at 2 years. Br J Ophthalmol, 2000; 84: 456–463. DOI: 10.1136/bjo.84.5.456 10781507 PMC1723467

[19] O’Connor, PM, Harper, CA and Brunton, CL. Shared care for chronic eye diseases: perspectives of ophthalmologists, optometrists and patients. Med J Australia, 2012; 196: 646–50. DOI: 10.5694/mja11.10856 22676881

[20] Tan, JC, Poh, EW, Srinivasan, S and Lim, TH. A pilot of tele-ophthalmology for diagnosis of chronic blurred vision. J Telemed Telecare, 2013; 19: 65–9. DOI: 10.1177/1357633x13476233 23520212

[21] Jenkinson, C, Coulter, A and Bruster, S. Patient’s experiences and satisfaction with health care: results of a questionnaire study of specific aspects of care. Qual Saf Health Care, 2002; 11: 335–9. DOI: 10.1136/qhc.11.4.335 12468693 PMC1757991

[22] Honeycutt, AA, Clayton, L and Khavyou, O. Guide to analysing the cost-effectiveness of Community Public Health Prevention Approaches N.C., USA: RTI International, 2006 Available from http://aspe.hhs.gov/health/reports/06/cphpa/report.pdf. Accessed 4 January 2015.

[23] Jones, B, Jarvis, P, Lewis, JA and Ebbutt, EF. Trials to assess equivalence: the importance of rigorous methods. BMJ, 1996; 313: 36 DOI: 10.1136/bmj.313.7048.36 8664772 PMC2351444

[24] Walker, E and Nowacki, AS. Understanding equivalence and noninferiority testing. J Gen Intern Med, 2010; 26: 192–6. DOI: 10.1007/s11606-010-1513-8 20857339 PMC3019319

[25] Dasgupta, A, Lawson, KA and Wilson, JP. Evaluating equivalence in noninferiority trials. Am J Health Syst Pharm, 2010; 67: 1337–43. DOI: 10.2146/ajhp090507 20689122

[26] Lui, KJ and Chang, KC. Notes on testing noninferiority in ordinal data under the parallel groups design. J Biopharm Stat, 2013; 23: 1294–1307. DOI: 10.1080/10543406.2013.834923 24138433

[27] Agresti, A. Generalized odds ratios for ordinal data. Biometrics, 1980; 36: 59–67. DOI: 10.2307/2530495

[28] Van der Schoot, J, Reus, NJ and Garway-Heath, DF. Accuracy of matching optic discs with visual fields: the European Structure and Function Assessment Trial (ESAFAT). Ophthalmol, 2013; 120: 2470–5. DOI: 10.1016/j.ophtha.2013.05.026 23809273

[29] Cheah, J. Chronic disease management: a Singapore perspective. BMJ, 2001; 393: 990–3. DOI: 10.1136/bmj.323.7319.990 PMC1121513 11679392

[30] Lim, AY, Tan, CS, Lau, TC, Tan, TL, Goh, LH and Teng, GG. Integrating rheumatology care in the community: can shared care work? Int J Integr Care, 2015; 19: e031 DOI: 10.5334/ijic.1990 PMC4548709 26312059

