# Supplementary figures and images for: Shared Care for Patients with Diabetes at Risk of Retinopathy: A Feasibility Trial
Source: Int J Integr Care. 2019 Sep 18;19(3):18. doi: 10.5334/ijic.4208 (PMC6753306; doi:10.5334/ijic.4208)

## Appendix 1. Recruitment of study patients

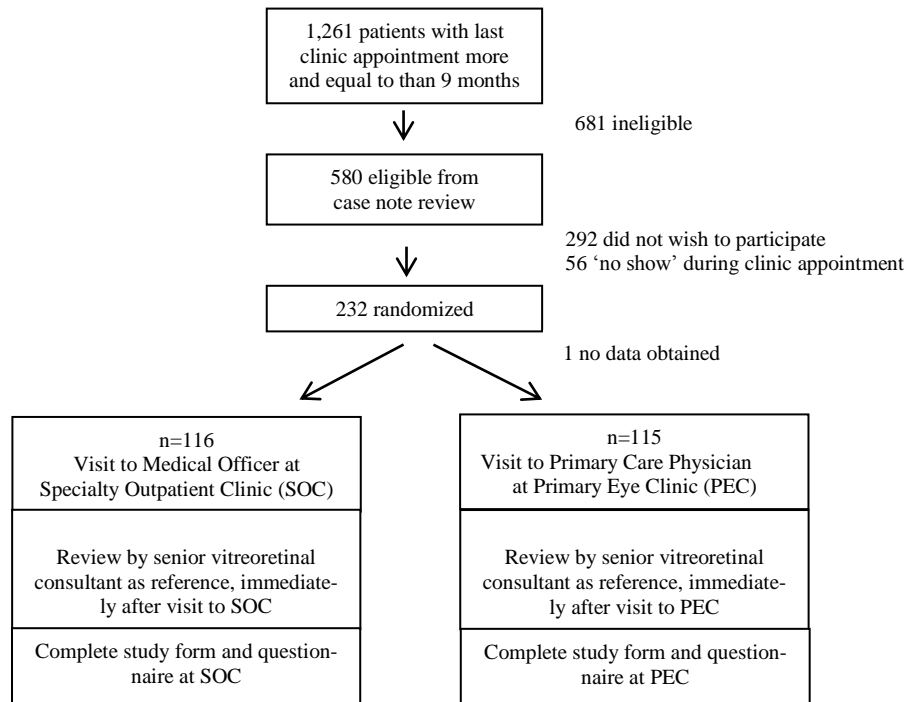

Supplement: Appendix 1. — Recruitment of study patients. [file ijic-19-3-4208-s1.pdf]
