# Supplementary material for: Shared Care for Patients with Diabetes at Risk of Retinopathy: A Feasibility Trial
Source: Int J Integr Care. 2019 Sep 18;19(3):18. doi: 10.5334/ijic.4208 (PMC6753306; doi:10.5334/ijic.4208)
Supplement: Appendix 2. — Clinical Pathways for referral of patients with DR from Specialist Eye Clinic (SOC) to Primary Eye Care Clinic (PEC). [file ijic-19-3-4208-s2.pdf]

Appendix 2. Clinical Pathways for referral of DR patients from Specialist Eye Clinic (SOC) to Primary Eye Care Clinic (PEC)

PATIENT UNDER D.R.S. FOLLOW-UP
FOR AT LEAST 2 YEARS

Exclusion Criteria (Not To Refer To PEC)

1. Ocular Co-Morbidity: Other Subspecialty Follow Up At SNEC
2. Diabetic Maculopathy (As Defined)
3. Require Drs Follow Up ≤ 8 Months
4. Unstable/Progressive Worsening Visual Acuity On Drs Follow Ups
5. One Eyed Patient (good eye VA less than 6/18 OR any eye with visual field of less than 10 degree)
6. Lens Opacity Classification: ≥ No4, Nc4 And/Or C5 And /Or P4 prevents fundus exam

REFER BACK TO GENERAL CLINIC

- No Diabetic Retinopathy or Maculopathy
- No Cataract/Post-Cataract Surgery
- No Ocular Co-Morbidity
- Vision Better or Equal to 6/9

REFER to PEC

Must be vetted by
Associate Consultant and above

Inclusion criteria for one eye patients:
good eye with VA better or equal to 6/18

NO DIABETIC RETINOPATHY

- Vision Better Or Equal To 6/36
- Media Opacity: < No4, No4, NC4, C5 and/or P4
- No Exclusion Criteria

MILD DIABETIC RETINOPATHY

- At Least One Microaneurysm
- Criteria For Moderate/Severe/Proliferative D.R. Not Met
- Vision Better Than Or Equal To 6/12
- No Exclusion Criteria

STABLE DIABETIC RETINOPATHY

- Post-PRP Burnt-Out Retinopathy
- No Maculopathy
- No Visual Deterioration For At Least One Year
- Clear Media
- No Exclusion Criteria
