# Supplementary material for: Shared Care for Patients with Diabetes at Risk of Retinopathy: A Feasibility Trial
Source: Int J Integr Care. 2019 Sep 18;19(3):18. doi: 10.5334/ijic.4208 (PMC6753306; doi:10.5334/ijic.4208)
Supplement: Appendix 3. — DR Clinical Diagnosis & Management Checklist. [file ijic-19-3-4208-s3.pdf]

Appendix 3. DRS Clinical Diagnosis & Management Checklist

**Date**<br>**HbA1c :** **%** **Test Date:**<br>**VA: VR:** **VL:**<br>**IOP (GAT): RE:** **mm Hg** **LE:** **mm Hg**	**Patient ID**

HISTORY

☐ Diabetes mellitus, Type ☐ 1 ☐ 2, Duration:<br>Treatment: ☐ Diet ☐ Oral Agents ☐ Insulin		
☐ Hypertension	☐ Dyslipidemia	☐ Heart Disease
☐ Nephropathy	☐ Neuropathy	☐ Others, please specify:
**Complaint/ Nature of the visit**:		

EXAMINATIONS

		RIGHT EYE	LEFT EYE
1	**Any significant changes in VA (≥2 lines)**	☐ Yes ☐ No	☐ Yes ☐ No
2	**Vert Cup Disc Ratio**	☐ • ☐ ☐	☐ • ☐ ☐
3.1	**Diabetic Retinopathy (No previous treatment)**	☐ None ☐ Mild<br>☐ Moderate ☐ Severe ☐ PDR	☐ None ☐ Mild<br>☐ Moderate ☐ Severe ☐ PDR
	**OR**		
3.2	**Previous Treatment**	☐ PRP ☐ Focal	☐ PRP ☐ Focal
	**3.2.1 Retinopathy status**	☐ Stable (Quiescent)<br>☐ Unstable (Worsening / New complications)	☐ Stable (Quiescent)<br>☐ Unstable (Worsening / New complications)
4	**Any macular edema?**<br>**If yes,**	☐ Yes ☐ No<br>☐ CSME<br>☐ Not clinically significant	☐ Yes ☐ No<br>☐ CSME<br>☐ Not clinically significant
5	**Other complications**	☐ Vitreous haemorrhage<br>☐ Increased retinal detachment<br>☐ Others : ________________________	☐ Vitreous haemorrhage<br>☐ Increased retinal detachment<br>☐ Others : ________________________

**CATARACT**<br>☐ Yes ☐ No<br>Keen for surgery<br>☐ Yes ☐ No	**Lens Status**	Phakic ☐<br>Pseudophakic ☐<br>Aphakic ☐	Phakic ☐<br>Pseudophakic ☐<br>Aphakic ☐
	**LOCS III Grading**	**NS_____ / C_____ / PSC_____**	**NS_____ / C_____ / PSC_____**

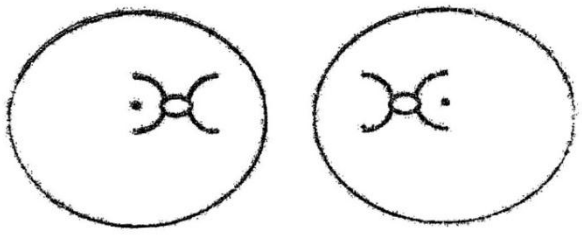

**PRELIMI-NARY TCU**	**PEC**<br>☐ 9 months<br>☐ 12 months<br>☐ 18 months	**SOC**<br>☐ < 1 week<br>☐ 1 to 4 weeks<br>☐ 5 to 12 weeks<br>☐ 3 to 6 months

CONSULTANT TO COMPLETE: FINAL ASSESSMENT

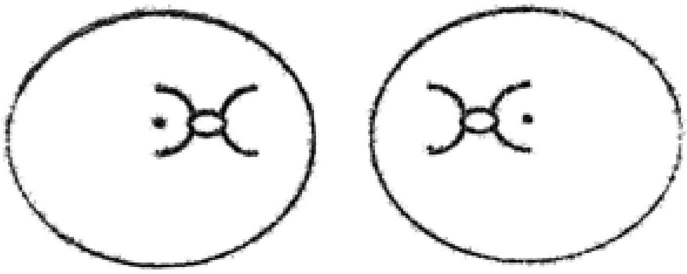

FINAL TCU	**PEC**<br>☐ 9 months<br>☐ 12 months<br>☐ 18 months	**SOC**<br>☐ < 1 week<br>☐ 1 to 4 weeks<br>☐ 5 to 12 weeks<br>☐ 3 to 6 months

Examining doctor **diagnosis**	Examining doctor **management**
☐ Satisfactory<br>☐ Unsatisfactory	☐ Satisfactory<br>☐ Unsatisfactory

Stamp, Signature and Date
