# Supplementary material for: Shared Care for Patients with Diabetes at Risk of Retinopathy: A Feasibility Trial
Source: Int J Integr Care. 2019 Sep 18;19(3):18. doi: 10.5334/ijic.4208 (PMC6753306; doi:10.5334/ijic.4208)
Supplement: Appendix 4. — Characteristics of patients with diabetes at risk of DR in PEC and SOC. [file ijic-19-3-4208-s4.pdf]

Appendix 4. Characteristics of patients with stable DR in PEC and SOC

	**PEC**<br>N=115	**SOC**<br>N=116	p
Age	65.7 (+/-8.7)	62.8 (+/-8.8)	0.013
Female	51.3%	59.5%	0.21
Chinese race	80.0%	83.6%	0.475
Married	81.5%	75.5%	0.329
Type 2 DM	75.7%	91.4%	0.001
Rx –oral agent for DM	58.3%	80.2%	<0.001
HbA1c	7.42 (+/-1.12)	7.14 (+/-1.15)	0.147
Secondary and below education	72.8%	79.6%	0.295
Occupation – not working	56.8%	46.9%	0.187
Income less than S$2,000/month	53.1%	29.6%	0.002
Housing – 1-3 room HDB	24.7%	28.6%	0.558
Never smoking	90.1%	88.8%	0.769
Never alcohol	91.4%	86.7%	0.319

PEC, Primary Eye Care Clinic; SOC, Specialist Outpatient Clinic; DM, Diabetes Mellitus; HDB, House Developing Board Singapore
