# Supplementary material for: Shared Care for Patients with Diabetes at Risk of Retinopathy: A Feasibility Trial
Source: Int J Integr Care. 2019 Sep 18;19(3):18. doi: 10.5334/ijic.4208 (PMC6753306; doi:10.5334/ijic.4208)
Supplement: Appendix 5. — Disconcordance in clinical assessment and or management with gold standard: potential impact on patients. [file ijic-19-3-4208-s5.pdf]

Appendix 5. Disconcordance in clinical assessment and or management with gold standard: potential impact on patients

	PEC (7 cases out of 115)	SOC (13 cases out of 116)
**Potential impact of disconcordance**		
No impact	4.39% (5)	7.76% (9)
Potentially mild impact	1.75% (2)	2.59% (3)
Potentially significant impact	-	0.86% (1)
